# Live Multi-Strain Probiotics Enhance Growth Performance by Regulating Intestinal Morphology and Microbiome Population in Weaning Piglets

**DOI:** 10.3390/microorganisms12112334

**Published:** 2024-11-15

**Authors:** Chao-Wei Huang, Shi-Yong Liu, Bishnu Prasad Bhattarai, Ting-Yu Lee, Hsiao-Tung Chang, Hsiao-Ching Lin, Hsiu-Ming Weng, Hsin-Hsuan Huang, Jin-Seng Lin, Jai-Wei Lee

**Affiliations:** 1 Department of Tropical Agriculture and International Cooperation, National Pingtung University of Science and Technology, Pingtung 91201, Taiwan; cwhuang@mail.npust.edu.tw (C.-W.H.); liup372@gmail.com (S.-Y.L.); bbhattarai987@gmail.com (B.P.B.); 2 SYNBIO TECH Inc., Kaohsiung 821011, Taiwan; tingyu.lee@synbiotech.com.tw (T.-Y.L.); hsiaotung@synbiotech.com.tw (H.-T.C.); lhc312@synbiotech.com.tw (H.-C.L.); hsiumingw@synbiotech.com.tw (H.-M.W.); thera_huang@synbiotech.com.tw (H.-H.H.); jslin@synbiotech.com.tw (J.-S.L.)

**Keywords:** probiotics, intestinal integrity, gene expression, growth performance, weaning piglets

## Abstract

The effects of different forms of multi-strain probiotics on weaning piglets are limitedly addressed. Thus, this study investigated the effects of live or inanimate multi-strain probiotics comprising *Lactobacillus plantarum*, *Streptococcus thermophilus*, and *Bacillus subtilis* on growth performance, intestinal morphology, fecal microbiota, short-chain fatty acids, and intestinal gene expression of weaning piglets. A total of 160 weaning piglets (4 weeks old) were randomly allocated into four treatments (CON: basal diet; AB: basal diet with 110 ppm and 66 ppm colistin in the weaning and nursery phases, respectively; LP: basal diet with 2.0 × 10^9^ CFU/kg live probiotics; and IP: basal diet with 2.0 × 10^9^ CFU/kg inanimate probiotics). Piglets fed with LP had significantly lower FCR compared to those of the CON and IP groups in week 4 to week 8 (*p* < 0.05). Moreover, the LP group had significantly higher villus height (VH) compared with AB at week 6, lower crypt depth (CD) compared with IP, and higher VH/CD ratio compared to other treatments at week 10 (*p* < 0.05), which indicate healthier intestinal morphology. Probiotic treatments (LP and IP) increased *Bifidobacterium* population compared to CON at week 6 and lowered *Enterobacteriaceae* at week 6 and week 10 (*p* < 0.05). Regarding gene expressions of intestinal integrity, LP showed significantly higher TFF3 expression compared with CON and AB at week 6 and compared with other treatments in jejunum at week 10 (*p* < 0.05). IP treatment had significantly higher MUC2 expression compared to other treatments at week 6 and week 10 (*p* < 0.05). Overall, live multi-strain probiotics improved growth efficiency by enhancing gut integrity and microbiome balance, making them a potential antibiotic alternative to ameliorate weaning stress and promote productive performance in weaning piglets.

## 1. Introduction

Weaning is a critical stage, involving multiple stressors such as dietary changes, abrupt separation from the sows, physical handling, transportation, and the establishment of social hierarchy, all of which induce weaning stress [1]. Weaning stress poses a significant threat to pig production, causing gastrointestinal disorders, diarrhea, impaired immunity, and even sudden death in piglets during the first two weeks after weaning, and surviving piglets may experience growth retardation. Post-weaning diarrhea (PWD) is one of the common intestinal disorders primarily caused by Gram-negative bacteria, such as *Escherichia coli*, *Campylobacter* spp., and *Salmonella* spp., along with weaning stress that negatively affect the gut integrity and growth performance of piglets [2,3,4]. The incidence of PWD was reported to be around 30% in the US and 24% in Australia, and the mortality rate was around 20 to 30% among infected piglets in 2017 [1,5]. Thus, PWD emerges as a major health and management problem in pig weaning operation. To improve production efficiency, antibiotic growth promoters (AGPs) are supplemented in piglet diets to inhibit the growth of pathogenic bacteria in the intestine and prevent weaning-associated disorders [6,7]. However, several countries have restricted AGP administration in animal diets as it could result in antibiotic-resistant pathogens and environmental contamination [8,9]. Therefore, numerous natural feed additives have been investigated to replace or reduce the use of AGPs. 

Probiotics are live microorganisms that, when ingested in adequate amounts, confer positive functions on host health due to their ability to provide intestinal microbial balance [10,11]. Probiotics modulate microbiota via competitive exclusion by adhesion sites and substrates and inhibit pathogens through the production of antimicrobial peptides and reduction of luminal pH [12,13]. In addition, probiotics also enhance gut-associated immunity by regulating mucus secretion and expression of tight junction proteins, inducing anti-inflammatory cytokines and reducing pro-inflammatory cytokines [14]. The diverse modes of action of probiotics also include stimulation of the production of epithelium-protective substances, such as mucins and their co-expressed Trefoil factor family (TFF) types [15,16,17,18]. The intestinal mucin layer constitutes mucins (MUCs) that form an intestinal chemical barrier that impairs bacteria on the epithelial surface through its polymeric structure and a high level of glycosylation [19]. Moreover, TFF peptides are expressed in intestinal epithelial cells and hold fundamental functions in the epithelial restoration network [18]. Thus, after also assessing the influence of probiotics on physical and chemical intestinal barrier integrity, such as tight junctions, mucin production was considered.

Currently, various strains of microorganisms are being examined as probiotics in animal production. Nevertheless, lactic acid bacteria, such as *Lactobacillus*, *Streptococcus*, and *Bifidobacteria* along with *Bacillus* species, are the commonly used species [20,21]. *Lactobacillus plantarum* (*L. plantarum*) is a Gram-positive lactic acid bacterium that is widely distributed in the gastrointestinal tract of animals and humans [22]. They are known to ferment carbohydrates to produce lactic acids, exhibit tolerance against bile salts, function under low pH, and compete with intestinal pathogens, such as *Enterobacteriaceae* [23,24]. Moreover, *Streptococcus thermophilus* (*S. thermophilus*) is also a Gram-positive lactic acid bacterium, commonly found in dairy products, and possesses numerous functional activities, including production of extracellular polysaccharides, bacteriocins, and vitamins [25]. *Bacillus subtilis* (*B. subtilis*) is a Gram-positive, rod-shaped, spore-forming bacterium [26]. Thus, their spore-forming ability gives them a long shelf life and resistance against adverse environmental conditions, such as acidic environment in the gastrointestinal tract. However, age of the animals, feed composition, feed preparation methods, and strains, forms, and concentration of probiotics are some factors that affect the efficacy of probiotics on the host [27,28].

The urge to enhance animal performance and reach the maximum genetic growth potential drives enhancing the efficacy of probiotics. The form of probiotics is an important factor that alters their functions. Live probiotics contain viable microorganisms and possess beneficial effects on host health upon ingestion, as they can colonize their host’s gut and restore the natural balance of the intestinal microbiome [29]. Wang et al. [30] summarized that live *L. johnsonii* probiotic at 1.0 × 10^9^ CFU/kg outperformed control and inactivated probiotics in improving growth performance, intestinal morphology, and microbial profile in broilers. On the other hand, inanimate probiotics are easier to handle and store, and they maintain some of their fundamental functions to improve animal performance without being affected by environmental conditions while providing longer shelf life [31,32]. Inactivated probiotics could extract some of their bacterial components, such as lipoteichoic acids and peptidoglycans that help to maintain their fundamental functions on the host [31]. Awad et al. [33] proposed that selecting more efficient probiotic strains and using multiple probiotics are some other measures to optimize the efficacy of probiotics. This statement is supported by the findings of Aiyegoro et al. [34], who summarized that supplementation of multi-strain probiotics significantly improved ADG, feed conversion rate (FCR), IgG concentration in blood serum, and lower enteric bacteria in the ileum when compared to those of piglets fed with a single-strain probiotic and a control diet (*p* < 0.05). 

Based on our knowledge, limited studies have addressed the effects of multi-strain probiotics in different forms on the growth performance and gut health of weaning piglets. Therefore, it was hypothesized that administration of multi-strain probiotics containing *L. plantarum*, *S. thermophilus*, and *B. subtilis* can improve intestinal health and enhance the growth performance of piglets. Thus, this study investigated the effects of live and inanimate multi-strain probiotics on growth performance, intestinal morphology, fecal microbiota, short-chain fatty acids production, and relative gene expression in crossbred (Landrace × Yorkshire × Duroc) weaning piglets.

## 2. Materials and Methods

### 2.1. Animals and Experimental Design

The trial was conducted in a commercial pig farm located in the southern part of Taiwan. A total of 160, mixed-sex crossbred piglets (Landrace × Yorkshire × Duroc) with an average body weight (BW) of 7.75 ± 0.02 kg weaned at 28 days of age were individually weighed and randomly allocated to 8 pens (n = 20 each, 10 barrows and 10 gilts) to have similar initial average body weight. The piglets were randomly divided into four different dietary treatments, two pens per treatment. Dietary treatments were basal diet (CON), basal diet with colistin at 110 ppm in the weaning phase and reduced to 66 ppm in the nursery phase (AB), basal diet with 2.0×10^9^ CFU/kg live multi-strain probiotics comprised of *L. plantarum*, *S. thermophilus*, and *B. subtilis* (LP), and basal diet with 2.0 × 10^9^ CFU/kg inanimate multi-strain probiotics comprised of *L. plantarum*, *S. thermophilus*, and *B. subtilis* (IP). The pigs were raised in semi-closed housing system for 6 weeks with an average temperature of 20.5 ± 4.2 °C and relative humidity of 73.9 ± 6.8%, respectively. The RespiSure-One vaccine (Zoetis, Parsippany-Troy Hills, NJ, USA) against *Mycoplasma hyopneumoniae* (P-5722-3, NL 1042), the Ingelvac CircoFLEX vaccine (Boehringer Ingelheim, Ridgefield, CT, USA) against porcine circovirus type 2 (PCV 2), and the Suigen HC vaccine (Vibrac, Taguig, Philippines) against attenuated swine fever virus, strain LPC-CN, were administrated at week 1, week 3, and week 6, respectively, following standard veterinary protocols. Feed and water were provided ad libitum. The protocols for the animal experiments in this research were approved by Institutional Animal Care and Use Committee (IACUC) of National Pingtung University of Science and Technology, Taiwan (NPUST 110-061). 

### 2.2. Experimental Diets

The multi-strain probiotics were purchased from a commercial company (SYNBIO TECH INC., Kaohsiung, Taiwan) and guaranteed to contain 2.0 × 10^9^ CFU/kg in both live and inanimate forms. The preparation of the inanimate multi-strain probiotic followed the modified method of Wagner et al. [35]. The probiotic powder was spread on sealed aluminum foil bags and autoclaved at 121 °C for 15 min. Aliquots were subsequently cultured on modified de Man Rogosa Sharpe (MRS) and plate count agar (PCA) plates. Serial dilution plate counts were performed before and after heat treatment to assess non-viability of inanimate multi-strain probiotics. Feed additives (colistin, live and inanimate muti-strain probiotics) were added into basal diet and prepared respective treatment diets in mash feed form. The experimental basal diets were based on corn soybean meal as illustrated in Table 1 and met or exceeded the nutritional requirements of pigs based on Council et al. [36]. The experimental diets were divided into two phases: weaning diet from week 4 to week 6 and nursery diet from week 6 to week 10.

### 2.3. Growth Performance

Piglets were weighed on electronic scale from week 4 to week 10 in two-week intervals to measure BW and ADG. In addition, feed intake was measured in each pen to calculate average daily feed intake (ADFI). The FCR was calculated using the ratio of total feed intake over total weight gain of a pen. 

### 2.4. Intestinal Morphology

Two piglets were randomly selected from each pen (n = 4 each treatment, with the body weight closest to the average body weight of the pen) and sacrificed for analyzing intestinal morphology, fecal microbiota, short-chain fatty acids production, and relative gene expression at week 6 and week 10, respectively. A section (2–3 cm) of samples was collected from the duodenum (15 cm from pylorus), the jejunum (15 cm from the ileocecal junction), and the ileum (5 cm proximal to the ileocecal junction) to evaluate intestinal morphology. The samples were gently flushed with 10% phosphate-buffered saline solution and stored in 10% buffered formalin. Each specimen was placed in an individual tissue cassette, embedded in paraffin wax which was followed by slicing at 3–4 μm thick sections and hematoxylin–eosin staining. A Nikon Eclipse E-200 microscope coupled with integrated camera and digital imaging analysis system (Nikon Digital System Fi1-L2, Nikon Co., Tokyo, Japan) was used for morphometric analysis of villus height (VH) from villus tip to villus–crypt junction, crypt depth (CD) from villus–crypt junction to lower limit of the crypt, and villus height to crypt depth (VH/CD) ratio [37,38].

### 2.5. Fecal Microbiota

Four pigs from each treatment were used for the collection of feces from rectum to assess probiotic effects on bacterial population at week 6 and week 10, respectively. Approximately 5 g of feces was collected and stored under −80 °C for further analysis [39,40]. Tenfold serial dilutions were prepared using 0.1 g of sample followed by centrifugation at 13,000× *g* for 5 min. Thereafter, 200 µL of precipitate was used for the genomic DNA extraction using the Genomic DNA Mini Kit (Geneaid Biotech LTD.) according to manufacturer’s instructions. The forward and reverse primer sequences for each bacterium are shown in Table 2, and the primer efficiency is shown in Table S1. An aliquot (1 µL) of cDNA template solution and 1 µL of each primer were mixed with 10 µL of SYBR Green which was further increased to total volume of 20 µL using DEPC-RNase free water. After preliminary denaturation, annealing and elongation were performed as described for Firmicutes, Bacteroidetes, Proteobacteria, and *Enterobacteriaceae* [41] and for *Bifidobacterium*, *Clostridium*, and *Lactobacillus* [42], followed by melting curve analysis on each sample. The resulting mixture was placed into a qPCR reactor (QuantStudio 3 real-time PCR systems, Thermo Fisher SCIENTIFIC, Waltham, MA, USA) for quantitative analysis, and Ct value was calculated with standard curve for the microbiota population (Log CFU/g).

### 2.6. Short-Chain Fatty Acids (SCFAs) Production

Short-chain fatty acids analysis (n = 4 for each treatment group) was performed using high-performance liquid chromatography instrument (SHIMADZU LC-40 HPLC System) equipped with a Rezex™ ROA-Organic Acid H+ (8%) column and conductometric detector (ICA-3030 TOA Electronics Co, Ltd., Tokyo, Japan). One gram of cecum content was mixed with 5 mL water, and 10% perchloric acid (KATAYAMA CHEMICAL Co., Ltd., Osaka, Japan) was added into it in a ratio of 9:1. The mixture was kept at 4 °C for 2 h and centrifuged at 10,000 rpm for 15 min. The supernatant was subsequently filtered with a 0.22 µm filter (Millipore Japan Ltd., Tokyo, Japan) followed by the injection of samples into the HPLC system. The column was eluted using 0.005N sulfuric acid solution as mobile phase A and a mixture of 0.005N sulfuric acid solution with 20 times molarity of Bis-Tris and 10 times molarity of ethylenediaminetetraacetic acid (EDTA) as mobile phase B. The solvent flow rate was set at 0.44 mL/min for each phase along with a column temperature of 40 °C. The analytical standards of all SCFAs were analyzed under the same conditions to establish standard calibration for quantitation of SCFAs in samples [43,44].

### 2.7. Real-Time Gene Expression

The gene expression of TFF2, TFF3, MUC2, ZO-1, Claudin 1, and Occludin was determined (n = 4 for each treatment group) using reverse transcription polymerase chain reaction (RT-PCR), and the primer efficiency is shown in Table S1. Firstly, 2 to 3 cm long jejunum samples were collected, washed with sterile saline solution to remove the digesta, and stored at −80 °C. The collected samples were used to extract the RNA using QIAGEN RNeasy^®^ Mini kit (74106). The RNA concentration was adjusted to 1 μg/μL to reverse transcribe the RNA into cDNA using High-Capacity cDNA Reverse Transcription kit with RNase inhibitor (4374966) under the conditions of 25 °C for 10 min further increased to 37 °C for 120 min and 85 °C for 5 min, and the reaction was cooled at 4 °C after completion. Glyceraldehyde-3-phosphate dehydrogenase (GAPDH) was used as housekeeping gene, and the primer sequences used in this analysis are illustrated in Table 2. The PCR efficiency was further checked according to the methods of Pfaffl (2001). The expression of selected genes was normalized to that of GAPDH for each treatment and expressed to the relative gene expression ratio based on amplification efficiency (E) and crossing point (CP), as follows:

$$\mathrm{Relative}\;\mathrm{gene}\;\mathrm{expression}\;\mathrm{ratio}=\frac{{\left(E_{\mathrm{target}}\right)}^{\left(\mathrm{Control}\;{\mathrm{CP}}_{\mathrm{target}\;}-\;\mathrm{treatment}\;{\mathrm{CP}}_{\mathrm{target}}\right)}}{{{(E}_{\mathrm{ref}})}^{{(\mathrm{Control}\;\mathrm{CP}}_{\mathrm{reference}}-{\mathrm{treatment}\;\mathrm{CP}}_{\mathrm{reference}})}}$$

### 2.8. Statistical Analysis

Data were analyzed using one way ANOVA of GraphPad Prism software version 9.4.1 (Boston, MA, USA) in a completely randomized block design. The Tukey multiple comparison test was carried out to determine the means, and Kruskal–Wallis test was used to compare the non-numerical parameters including percentage and ratios. Means were considered to be significantly different at *p* < 0.05. Results in Table 1, Table 2, Table 3, Table 4, Table 5, Table 6 and Table 7 are expressed as means of treatments and the pooled standard error of the mean (SEM).

## 3. Results

### 3.1. Growth Performance

No significant differences were observed in BW, ADG, and ADFI among the treatments in different stages and the overall period (week 4 to week 10) (Table 3). However, piglets fed with LP had significantly lower FCR compared to CON and IP groups in week 4 to week 6 (*p* < 0.05). In addition, a significantly better FCR was observed in LP compared to CON and IP in following week 6 to week 8 (*p* < 0.05). No significant difference regarding FCR was observed among the groups in week 8 to week 10 and the overall period (week 4 to week 10).

**Table 3:** Effects of live or inanimate multi-strain probiotics on the growth performance in weaning pigs ^1^.

Items ^2^	Treatments ^3^				SEM	*p*-Value
	CON	AB	LP	IP		
BW, kg/pig						
Week 4	7.73	7.72	7.74	7.71	0.01	0.999
Week 6	11.64	11.46	11.91	11.69	0.19	0.908
Week 8	18.57	18.90	19.40	19.01	0.34	0.950
Week 10	27.20	26.91	28.15	27.66	0.54	0.926
ADG, kg/pig						
Week 4–6	0.27	0.26	0.29	0.28	0.01	0.889
Week 6–8	0.49	0.54	0.53	0.52	0.02	0.836
Week 8–10	0.60	0.60	0.63	0.62	0.02	0.897
Week 4–10	0.47	0.46	0.49	0.48	0.01	0.926
ADFI, kg/pig						
Week 4–6	0.37	0.37	0.39	0.40	0.02	0.766
Week 6–8	0.75	0.73	0.77	0.76	0.02	0.974
Week 8–10	1.05	1.04	1.07	1.10	0.02	0.912
Week 4–10	0.71	0.71	0.74	0.75	0.02	0.998
FCR, kg/kg						
Week 4–6	1.41 ^a^	1.41 ^ab^	1.36 ^b^	1.49 ^a^	0.05	0.003
Week 6–8	1.56 ^a^	1.41 ^b^	1.43 ^b^	1.51 ^a^	0.07	<0.001
Week 8–10	1.75	1.74	1.71	1.76	0.02	0.088
Week 4–10	1.55	1.55	1.51	1.58	0.03	0.163

^1^ SEM, standard error of the mean. ^ab^ Mean values with different lowercase letters in the same row differ significantly at *p* < 0.05. ^2^ BW, body weight; ADG, average daily gain; ADFI, average daily feed intake; and FCR, feed conversion ratio. ^3^ CON, piglets were fed with basal diet; AB, piglets were fed with basal diet containing 110 ppm colistin in the weaning phase and 66 ppm colistin in the nursery phase; LP and IP, piglets were fed with basal diet containing 2.0 × 10^9^ CFU/kg live and inanimate multi-strain probiotics containing *L. plantarum*, *S. thermophilus*, and *B. subtilis*, respectively.

### 3.2. Intestinal Morphology

**Figure 1:**
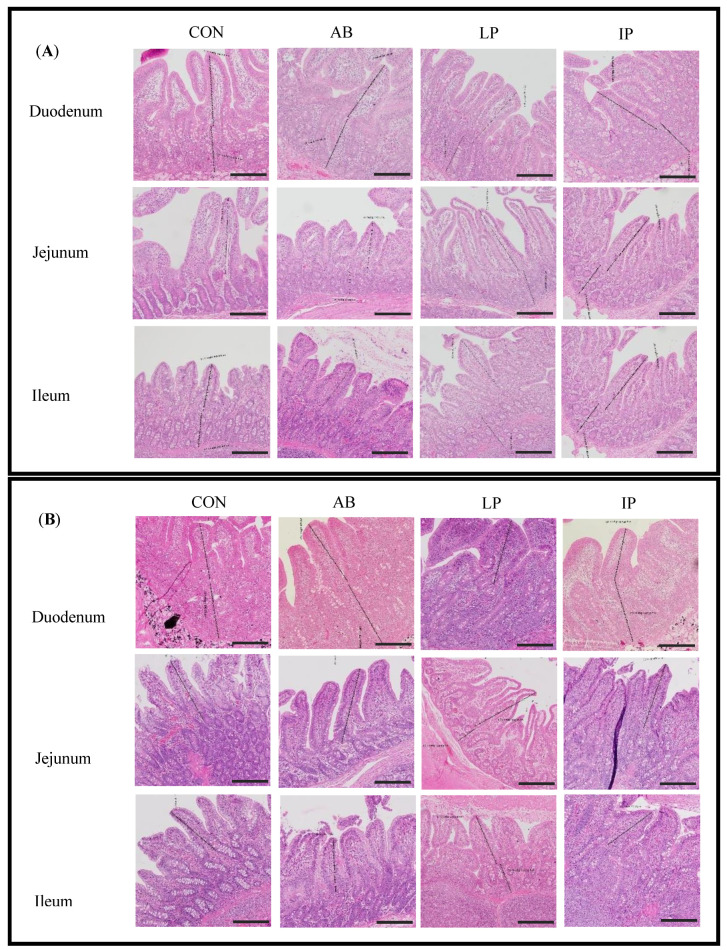
Histological representations of intestinal samples (duodenum, jejunum, and ileum) from experimental pigs at week 6 (**A**) and week 10 (**B**) (scale bar = 250 µm). CON, piglets were fed with basal diet; AB, piglets were fed with basal diet containing 110 ppm colistin in the weaning phase and 66 ppm colistin in the nursery phase; LP and IP, piglets were fed with basal diet containing 2.0 × 10^9^ CFU/kg live and inanimate multi-strain probiotics containing *L. plantarum*, *S. thermophilus*, and *B. subtilis*, respectively.

Supplementation of LP had significantly longer jejunal VH with respect to AB at week 6 (*p* < 0.05) (Table 4). However, no significant differences were observed in VH of the duodenum and ileum among the groups at week 6. Regarding CD, there was no significant difference in duodenum and jejunum among the treatments at week 6. Nevertheless, pigs fed LP had significantly higher ileal CD compared with IP at week 6 (*p* < 0.05). There were no significant differences in duodenal, jejunal, and ileal VH/CD ratio among the groups at week 6 (Table 4). At week 10, LP had significantly lower CD than IP in jejunum, and the VH/CD ratio was significantly higher than all the other groups (*p* < 0.05) (Table 5). No significant differences were found in the duodenal and ileal VH, CD, and VH/CD ratio among the groups. The histological representations of intestinal segments of 6-week-old pigs and 10-week-old pigs are presented in Figure 1A and Figure 1B, respectively. 

**Table 4:** Effect of live or inanimate multi-strain probiotics on intestinal morphology in 6-week-old weaning pigs ^1^.

Items ^2^	Treatments ^3^				SEM	*p*-Value
	CON	AB	LP	IP		
Duodenum						
Villus height, μm	505.81	458.91	496.67	525.80	14.02	0.655
Crypt depth, μm	324.69	363.57	296.88	353.41	15.04	0.166
VH/CD ratio	1.65	1.57	2.21	1.53	0.16	0.316
Jejunum						
Villus height, μm	519.09 ^a^	394.04 ^b^	512.52 ^a^	452.75 ^ab^	29.27	0.001
Crypt depth, μm	236.00	193.67	235.67	237.76	10.71	0.293
VH/CD ratio	2.28	2.42	2.26	2.16	0.05	0.837
Ileum						
Villus height, μm	362.62	382.43	370.29	373.93	4.11	0.928
Crypt depth, μm	242.84 ^ab^	269.77 ^a^	253.39 ^a^	188.33 ^b^	17.64	0.011
VH/CD ratio	1.56	1.67	1.54	2.04	0.12	0.094

^1^ SEM, standard error of the mean. ^ab^ Mean values with different lowercase letters in the same row differ significantly at *p* < 0.05. ^2^ VH/CD ratio, villus height to crypt depth ratio. ^3^ CON, piglets were fed with basal diet; AB, piglets were fed with basal diet containing 110 ppm colistin in the weaning phase and 66 ppm colistin in the nursery phase; LP and IP, piglets were fed with basal diet containing 2.0 × 10^9^ CFU/kg live and inanimate multi-strain probiotics containing *L. plantarum*, *S. thermophilus*, and *B. subtilis*, respectively.

**Table 5:** Effect of live or inanimate multi-strain probiotics on intestinal morphology in 10-week-old weaning pigs ^1^.

Items ^2^	Treatments ^3^				SEM	*p*-Value
	CON	AB	LP	IP		
Duodenum						
Villus height, μm	382.63	478.48	442.97	393.76	22.27	0.137
Crypt depth, μm	397.97	494.69	424.39	444.18	20.45	0.376
VH/CD ratio	1.09	1.44	1.16	0.96	0.10	0.251
Jejunum						
Villus height, μm	423.84	412.95	479.07	412.46	15.88	0.134
Crypt depth, μm	279.55 ^ab^	232.68 ^ab^	193.00 ^b^	303.41 ^a^	24.59	0.007
VH/CD ratio	1.64 ^b^	1.93 ^b^	2.67 ^a^	1.77 ^b^	0.23	0.001
Ileum						
Villus height, μm	396.72	367.76	362.94	390.19	8.28	0.689
Crypt depth, μm	298.54	290.42	209.3	298.35	21.70	0.097
VH/CD ratio	1.51	1.48	1.96	1.38	0.13	0.183

^1^ SEM, standard error of the mean. ^ab^ Mean values with different lowercase letters in the same row differ significantly at *p* < 0.05. ^2^ VH/CD ratio, villus height to crypt depth ratio. ^3^ CON, piglets were fed with basal diet; AB, piglets were fed with basal diet containing 110 ppm colistin in the weaning phase and 66 ppm colistin in the nursery phase; LP and IP, piglets were fed with basal diet containing 2.0 × 10^9^ CFU/kg live and inanimate multi-strain probiotics containing L. plantarum, S. thermophilus, and B. subtilis, respectively.

### 3.3. Fecal Microbiota

The relative populations of fecal microbiota of 6-week-old and 10-week-old pigs among treatments were compared (Table 6). Piglets fed with multi-strain probiotics (LP and IP) had significantly lower *Enterobacteriaceae* population compared to the CON group at week 6 (*p* < 0.05). In addition, the population of *Enterobacteriaceae* was significantly lower in the LP group with respect to CON at week 10. Moreover, multi-strain probiotics (LP and IP) had significantly higher *Bifidobacterium* population compared to CON and significantly lower compared to the AB group at week 6 (*p* < 0.05). In contrast, the *Bifidobacterium* population was significantly lower in LP with respect to other treatments at week 10. At week 10, the AB group had significantly lower Proteobacteria population compared to the CON group (*p* < 0.05). However, multi-strain probiotics supplementation did not alter Proteobacteria population compared to the other groups at both week 6 and week 10. 

**Table 6:** Effects of live or inanimate multi-strain probiotics on fecal microbiota in weaning pigs ^1^.

Items	Treatments ^2^				SEM	*p*-Value
	CON	AB	LP	IP		
Week 6 (Log_10_ copy number/g)						
Phylum						
Firmicutes	9.93	9.84	9.86	9.73	0.172	0.675
Bacteroides	9.75	10.06	9.83	9.77	0.316	0.715
Proteobacteria	7.84	6.96	6.58	6.75	0.42	0.082
Family						
*Enterobacteriaceae*	7.62 ^a^	6.53 ^b^	6.11 ^b^	6.29 ^b^	0.352	0.005
Genus						
*Bifidobacterium*	5.56 ^c^	7.54 ^a^	7.26 ^b^	6.59 ^b^	0.232	0.001
*Clostridium*	9.00	8.98	8.91	8.77	0.199	0.584
*Lactobacillus*	7.15	7.51	7.44	7.00	0.381	0.847
Week 10 (Log_10_ copy number/g)						
Phylum						
Firmicutes	9.97	9.97	9.91	9.96	0.073	0.819
Bacteroides	9.92	9.78	9.62	9.65	0.118	0.182
Proteobacteria	6.88 ^a^	5.27 ^b^	6.34 ^ab^	6.20 ^ab^	0.305	0.007
Family						
*Enterobacteriaceae*	6.49 ^a^	4.94 ^c^	5.32 ^bc^	6.09 ^ab^	0.266	0.034
Genus						
*Bifidobacterium*	7.18 ^a^	7.58 ^a^	6.20 ^b^	7.41 ^a^	0.294	0.019
*Clostridium*	9.22	9.14	9.19	9.11	0.115	0.668
*Lactobacillus*	7.50	7.47	7.37	7.35	0.165	0.439

^1^ SEM, standard error of the mean. ^abc^ Mean values with different lowercase letters in the same row differ significantly at *p* < 0.05, which are statistically tested by Kruskal–Wallis test. ^2^ CON, piglets were fed with basal diet; AB, piglets were fed with basal diet containing 110 ppm colistin in the weaning phase and 66 ppm colistin in the nursery phase; LP and IP, piglets were fed with basal diet containing 2.0 × 10^9^ CFU/kg live and inanimate multi-strain probiotics containing *L. plantarum*, *S. thermophilus*, and *B. subtilis*, respectively.

### 3.4. SCFAs Production

The effects of different forms of multi-strain probiotics on SCFAs production in 6-week-old and 10-week-old pigs are presented in Table 7. The results illustrate that there were no significant differences in SCFAs production among the treatments at week 6 and week 10. 

**Table 7:** Effect of live or inanimate multi-strain probiotics on SCFAs production in weaning pigs ^1^.

Items ^2^	Treatments ^3^				SEM	*p*-Value
	CON	AB	LP	IP		
Week 6, mmol/g						
Acetic acid	7.67	8.58	7.83	6.85	0.355	0.734
Propionic acid	3.51	2.81	3.88	2.96	0.248	0.631
Butyric acid	1.39	2.08	2.12	1.72	0.171	0.405
Ace + Pro + Buty	12.57	13.47	13.83	11.53	0.514	0.559
Week 10, mmol/g						
Acetic acid	9.88	10.16	10.48	9.87	0.144	0.986
Propionic acid	4.65	4.33	5.07	5.02	0.173	0.900
Butyric acid	2.26	2.37	2.35	2.05	0.073	0.962
Ace + Pro + Buty	16.80	16.86	17.90	16.94	0.260	0.988

^1^ SEM, standard error of the mean. ^2^ Ace + Pro + Buty, combination of acetic acid, propionic acid, and butyric acid. ^3^ CON, piglets were fed with basal diet; AB, piglets were fed with basal diet containing 110 ppm colistin in the weaning phase and 66 ppm colistin in the nursery phase; LP and IP, piglets were fed with basal diet containing 2.0 × 10^9^ CFU/kg live and inanimate multi-strain probiotics containing *L. plantarum*, *S. thermophilus*, and *B. subtilis*, respectively.

### 3.5. Quantitative Real-Time PCR

**Figure 2:**
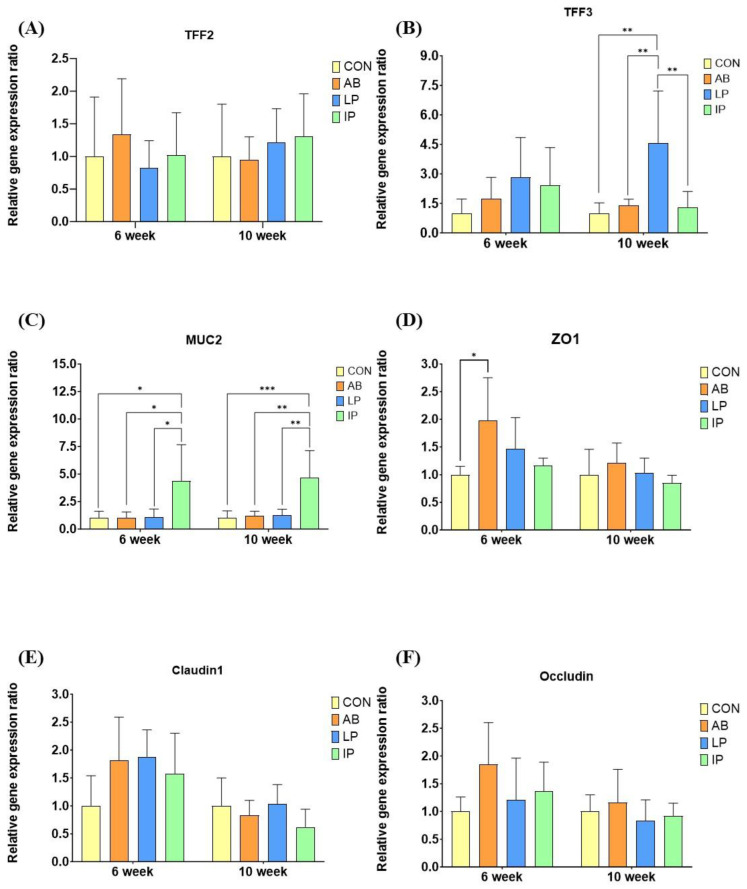
Effects of live or inanimate multi-strain probiotics on relative gene expression of weaning pigs. *, **, and *** indicate that means are significant difference among each other at *p* < 0.05, *p* < 0.01, and *p* < 0.001, respectively, which are statistically tested by Kruskal–Wallis test. (**A**) TFF2, Trefoil factor 2; (**B**) TFF3, Trefoil factor 3; (**C**) MUC2, Mucin 2; (**D**) ZO1, Zonula occludens-1; (**E**) Claudin 1; and (**F**) Occludin. CON, piglets were fed with basal diet; AB, piglets were fed with basal diet containing 110 ppm colistin in the weaning phase and 66 ppm colistin in the nursery phase; LP and IP, piglets were fed with basal diet containing 2.0 × 10^9^ CFU/kg live and inanimate multi-strain probiotics containing *L. plantarum*, *S. thermophilus*, and *B. subtilis*, respectively.

Dietary supplementation of multi-strain probiotics (LP and IP) did not affect the relative mRNA gene expression of molecular peptides (TFF2) and tight junction proteins (ZO-1, Claudin 1, and Occludin) compared with CON and AB at week 6 and week 10 (Figure 2). Pigs fed with both LP and IP had significantly higher TFF3 gene expression compared to CON and AB at week 6 (*p* < 0.001). At week 10, the expression level of TFF3 of LP remained significantly higher than all the other treatments (*p* < 0.0001). The MUC2 mRNA expression of IP was significantly higher than all the other groups at both week 6 and week 10 (*p* < 0.001).

## 4. Discussion

It has been an area of concern to discover sustainable and efficient alternatives for replacing antibiotic growth promoters in the pig industry. Studies have documented that supplementation of probiotics can regulate the intestinal microflora and improve the immunity and growth of piglets [45,46,47]. However, the effects of multi-strain probiotics, both in live and inanimate forms, on the gut health and production efficiency of weaning pigs have not been fully addressed. This study demonstrated that pigs fed with live multi-strain probiotics (LP) had significantly better FCR compared to CON and IP in week 4 to week 8 after weaning (*p* < 0.05). The intestinal microbiota of weaning piglets have not been fully developed, and microbiota stability is impaired in the first couple of weeks after weaning [48]. Therefore, probiotics are more effective in the early weaning stage during development or impairment of intestinal microbiota to balance the beneficial microbial population and maintain the gut integrity of piglets [27]. This statement is supported by intestinal morphology findings, where LP had significantly higher jejunal VH compared to AB but significantly higher ileal CD compared to IP at week 6 (*p* < 0.05). The absorption of dietary compounds mainly takes place in the proximal part of the small intestine [49]. Significantly higher jejunal VH in pigs supplemented with LP at week 6 could be associated with greater nutrient absorption capacity of the small intestine, resulting in higher energy intake and significantly better FCR in the early stages of weaning piglets (*p* < 0.05) [50]. Thus, these findings proposed that inclusion of LP in piglets’ diet could reduce the physiological changes induced by post-weaning stress in the small intestine and maintain healthier intestinal morphological structure in piglets at week 6. In agreement with our finding, Júnior, et al. [51] found that inclusion of live *B. subtilis* DSM 32540 increased the duodenal and jejunal VH/CD ratio and reduced the duodenal CD of weaning piglets (*p* < 0.05), which further resulted in significantly lower FCR in treated piglets two weeks after weaning. In addition, pigs fed with LP showed significantly lower jejunal CD compared with that of IP and significantly higher jejunal VH/CD ratio compared with that of other treatments at week 10 (*p* < 0.05). However, no significant differences were observed in overall BW, ADG, and ADFI among the treatments. The utilization of absorbed nutrients is affected by various factors including the environment, animal physiology, and animal diet. Thus, the increased availability of nutrients with improved intestinal morphology could be associated with maintaining homeostasis in response to environmental conditions, regulating basal physiological functions and strengthening the immune system, which might have resulted in minimal effects on the growth performance of 10 weeks old pigs. It was reported that weaning piglets fed with *B. licheniformis*-fermented feed additive, which contain probiotics and antimicrobial substances, had no significant differences in overall BW, ADG, ADFI, and FCR among the groups [52]. Lin and Yu [53] also concluded that probiotics did not alter overall growth performance, such as BW, ADG, ADFI, and FCR, compared with the control in weaning piglets. 

The present study shows that Firmicutes, Bacteroidetes, and Proteobacteria are the main dominant phyla of the total gut microbiome. Consistent with our finding, previous studies have reported that the most abundant taxa were the phyla Firmicutes, Bacteroidetes, and Proteobacteria of the total gut microbiota in weaning piglets [2,54]. The population of *Enterobacteriaceae* was significantly lower in all treatment groups compared to CON at week 6 and was significantly lower in LP treatments compared to the CON group at week 10. *Enterobacteriaceae* are Gram-negative bacteria that are causative agents of diarrhea, urinary tract infections, and other metabolic diseases in pigs [55,56]. Pupa et al. [57] concluded that there is a significant reduction in enterobacterial counts with the supplementation of probiotics, *L. plantarum*, and antibiotics in neonatal LYD pigs. In addition, Wang et al. [30] investigated the effects of live or disrupted *L. johnsonii* strain BS15 in broilers and found that both groups fed with live and disrupted probiotics significantly reduced *Enterobacteriaceae* compared to the control, which is in agreement with our result (*p* < 0.05). Furthermore, multi-strain probiotics (LP and IP) resulted in a significant increase in *Bifidobacterium* population compared to CON at week 6. These findings suggest that the crucial role of multi-strain probiotics is the inhibition of harmful bacteria and modulation of beneficial bacteria, thereby maintaining a balanced gut microbiota population. Dietary *B. subtilis* DSM 32315 was found to increase the abundances of ileal *Bifidobacterium* in comparison with the control in weaning piglets (*p* < 0.05) [54]. A study investigating the effects of live compound probiotics (*L. plantarum* and *S. cerevisiae*) in weaned piglets reported a significant increment in *Bifidobacterium* and *Lactobacillus* abundance in the treatment group [58]. In contrast, LP significantly lowered the *Bifidobacterium* population with respect to other treatments at week 10. Moreover, there were no significant differences in *Lactobacillus* and other bacterial populations among treatments at week 6 and week 10. The sensitivity of probiotics to adverse environmental conditions could be a contributing factor to the lack of change in the *Lactobacillus* population in the gastrointestinal tract. Veljović et al. [59] assessed the survival of *L. helveticus* BGRA43, *L. fermentum* BGHI14, and *S. thermophilus* BGVLJ1-44 in the simulated gastrointestinal tracts of sow and found that these probiotic strains adequately survived the passage through the stomach. In addition, only *L. helveticus* BGRA43 and *S. thermophilus* BGVLJ1-44 sustained the duodenal passage with a survival rate of approximately 10%, and they minimally survived conditions simulating the colon environment with a survival rate of 1 to 2% [59]. Despite alteration in the bacterial population, our study indicated that there were no significant differences in acetic acid, propionic acid, and butyric acid production among treatments at week 6 and week 10. While SCFAs production is a crucial factor in gut health, the overall benefits of probiotics supplementation on gut integrity and growth performance should not be underestimated. Thus, further studies need to be conducted to assess the viability of multi-strain probiotics in the intestinal tract of piglets to understand the interaction between probiotics, alterations in intestinal microbiota, SCFAs production, and the physiological status of piglets.

Piglets fed with LP and IP exhibited significantly higher TFF3 expression, which is related to gut integrity, compared to those fed with both CON and AB at week 6 (*p* < 0.001). In addition, LP continued to maintain its function of TFF3 expression in the jejunum compared to other treatments at week 10 (*p* < 0.0001). TFF3 can stimulate various signaling pathways, such as MAPK, NF-κB, PI3K, STAT3, mTOR, and HIF-1α, that could repair damaged mucosa and regulate lipid and glucose metabolism [60]. On the other hand, the mRNA expression of MUC2 in the jejunum was found to be significantly higher in IP compared to the other treatments at week 6 and week 10 (*p* < 0.001). This suggests that inanimate multi-strain probiotics outperformed to promote mucin secretion and maintain the intestinal mucosal integrity. The lipoteichoic acid found in the cell wall of lactic acid bacteria stimulates MUC2 expression by modulating the TLR2/p38 MAPK/NK-κB pathway, thereby preventing intestinal inflammation [61]. Piqué et al. [29] summarized that inanimate probiotics could extract bacterial components, such as lipoteichoic acids and peptidoglycans, which are important to maintain their fundamental characteristics. In addition, an increase in goblet cell number with an inclusion of probiotics promoted secretion of glycoproteins, including MUC2 by regulating the activation of the NF-κB pathway, and improved the mucus layer structure [62,63]. Taken together, our results indicated that both LP and IP were able to regulate different gene expressions in the intestine and constitute the first line of defense at the intestinal barrier. Nevertheless, it is also crucial to consider the potential implications on nutrients absorption. A significant increase in MUC2 gene expression could result in a denser mucosal layer, which may impede the efficient absorption of nutrients [64]. However, in our study, the supplementation of IP did not negatively affect the growth performance of piglets, suggesting that the upregulation of MUC2 gene expression may not affect nutrient absorption. No significant differences were observed in the expression of other genes related to gut integrity, including TFF2, ZO1, Claudin 1, and Occludin among the groups. In contrast to our findings, supplementation of multiple probiotics containing *B. subtilis* and *L. plantarum* significantly enhanced the mRNA expression levels of ZO-1 in the jejunum of pigs (*p* < 0.05) [65]. Júnior et al. [51] also concluded that inclusion of *B. subtilis* DSM 32540 significantly improved intestinal morphology and growth performance compared to the control (*p* < 0.05) but did not alter the gene expression of inflammatory markers, tight junction proteins, and SCFA transporters. Limited studies have addressed the effects of probiotics on gut-associated proteins in piglets. Moreover, supplementation of different strains, different forms, and different species of probiotics could be possible reasons to observe the inconsistent results of using probiotics on the performance of pigs over the past years. Therefore, further studies should be conducted considering all these factors to understand the probiotics’ modes of action in host–microflora interactions.

## 5. Conclusions

Dietary supplementation of live multi-strain probiotics was beneficial for improving growth efficiency in a way equivalent to antibiotics supplementation in weaning piglets during the early stage after weaning. In addition, live probiotics positively influenced intestinal integrity, as evidenced by healthier intestinal morphology, favorable microbial compositions, and higher expression of gut integrity-related genes in weaning piglets. These results suggest that live multi-strain probiotics could be a promising alternative for replacing or reducing antibiotic administration to reduce weaning stress by modulating gut health and promoting growth efficiency in weaning piglets. However, the present study did not highlight the influence of multi-strain probiotics during the grower and finisher stages, which might yield different outcomes. Therefore, the current study lays the foundation for future study on the optimization of probiotics concentrations, and further exploration of the mechanisms and validation of multi-strain probiotics on pigs’ performance. Robust validation of the current findings through large-scale animal trials could be fundamental for its commercial application as a sustainable and promising additive to ameliorate weaning stress and promote production performance in weaning piglets.

## Figures and Tables

**Table 1:** Ingredients and chemical composition of basal diets (as fed basis, %).

Items	Week 4–6	Week 6–10
Cooked corn	55.68	21.25
Corn	-	42.21
Full fat soybean meal	9.90	0.65
Soybean meal, dehulled	-	14.75
Fermented soybean meal	9.90	10.00
Whey powder	7.43	2.75
Fat powder	4.95	2.75
Soybean oil	1.68	4.05
Plasma meal	3.96	-
Fish meal	-	0.25
Calcium dihydrogen phosphate	0.79	0.07
Limestone	0.79	0.08
Humic acid	0.50	-
Acidifier	0.30	0.02
Emulsifier	0.05	-
Peptides	2.47	1.00
Phytase	0.03	-
Salt	0.10	0.04
Choline chloride 50%	0.15	0.01
Lysine 78%	0.40	0.04
Methionine 98%	0.20	0.01
Threonine 98%	0.10	0.01
Tryptophan %	0.08	0.01
Valine	-	0.03
Vitamins and Minerals	0.54	0.02
Total	100.00	100.00
Calculated composition	-	-
Digestible energy, MJ/kg	14.59	14.73
Metabolic energy, MJ/kg	14.46	14.38
Crude protein, %	18.60	19.23
Crude fat, %	8.42	7.44
Crude fiber, %	2.19	2.12
Crude ash, %	2.30	4.59
Lysine, %	1.35	1.25
Methionine + cystine, %	0.93	0.87
Calcium, %	0.70	0.72
Available phosphorus, %	0.46	0.40

**Table 2:** Forward and reverse primer sequences used for quantitative analysis of microbiota.

Samples ^1^	Primer	Gene Sequence
Strains		
Firmicutes	Forward	GGAGYATGTGGTTTAATTCGAAGCA
	Reverse	AGCTGACGACAACCARGCAC
Bacteroidetes	Forward	GGARCATGTGGTTTAATTCGATGAT
	Reverse	AGCTGACGACAACCATGCAG
Proteobacteria	Forward	CATGACGTTACCCGCAGAAGAA
	Reverse	CTCTACGAGACTCAAGCTTGC
*Bifidobacterium*	Forward	GGGTGGTAATGCCGGATG
	Reverse	TAAGCGATGGACTTTCACACC
*Enterobacteriaceae*	Forward	CATTGACGTTACCCGCAGAAGAAGC
	Reverse	CTCTACGAGACTCAAGCTTGC
*Clostridium*	Forward	CGGTACCTGACTAAGAAGC
	Reverse	AGTTTYATTCTTGCGAACG
*Lactobacillus*	Forward	AGCAGTAGGGAATCTTCCA
	Reverse	CACCGCTACACATGGAG
Gene Expression		
TFF2	Forward	ATCACCAGCGACCAGTGCTT
	Reverse	ATGACGCACTCCTCAGACTCTTG
TFF3	Forward	CAGGATGTTCTGGCTGCTAGTG
	Reverse	GCAGTCCACCCTGTCCTTG
MUC2	Forward	CAACGGCCTCTCCTTCTCTGT
	Reverse	GCCACACTGGCCCTTTGT
ZO-1	Forward	AAGCCCTAAGTTCAATCACAATCT
	Reverse	ATCAAACTCAGGAGGCGGC
Occludin	Forward	TCCTGGGTGTGATGGTGTTC
	Reverse	CGTAGAGTCCAGTCACCGCA
Claudin 1	Forward	AGAAGATGCGGATGGCTGTC
	Reverse	CCCAGAAGGCAGAGAGAAGC
GAPDH	Forward	AAGGAGTAAGAGCCCCTGGA
	Reverse	TCTGGGATGGAAACTGGAA

^1^ TFF2, Trefoil factor 2; TFF3, Trefoil factor 3; MUC2, Mucin 2; ZO-1, Zonula occludens-1; GAPDH, glyceraldehyde-3-phosphate dehydrogenase.

## Data Availability

All experimental data supporting the findings of this study are available from the corresponding author upon request. The data are not publicly available due to privacy.

## Supplementary Materials

The following supporting information can be downloaded at: https://www.mdpi.com/article/10.3390/microorganisms12112334/s1, Table S1. PCR efficiencies of analyzed parameters in the experiment.
