# Supplementary material for: Live Multi-Strain Probiotics Enhance Growth Performance by Regulating Intestinal Morphology and Microbiome Population in Weaning Piglets
Source: Microorganisms. 2024 Nov 15;12(11):2334. doi: 10.3390/microorganisms12112334 (PMC11596860; doi:10.3390/microorganisms12112334)
Supplement: Supplementary file 1 [file microorganisms-12-02334-s001.zip › microorganisms-3299305-supplementary.pdf]

Table S1. PCR efficiencies of analyzed parameters in the experiment.

| Items | Efficiency by $10^{(-1/slope)}$ |
|---|---|
| Firmicutes | 2.01 |
| Bacteroides | 2.04 |
| Proteobacteria | 1.98 |
| *Enterobacteriaceae* | 2.00 |
| *Bifidobacterium* | 2.02 |
| *Clostridium* | 1.88 |
| *Lactobacillus* | 2.04 |
| ZO-1 | 1.86 |
| OCC | 2.07 |
| Claudin 1 | 1.81 |
| MUC2 | 1.97 |
| TFF2 | 2.03 |
| TFF3 | 1.92 |
| GAPDH | 2.02 |
